# Supplementary material for: Reactive sputtering of SnS thin films using sulfur plasma and a metallic Tin target: achieving stoichiometry and large grains
Source: Sci Rep. 2025 Aug 7;15:28917. doi: 10.1038/s41598-025-14093-x (PMC12332127; doi:10.1038/s41598-025-14093-x)
Supplement: Supplementary file 1 — Supplementary Material 1 [file 41598_2025_14093_MOESM1_ESM.pdf]

Supplementary Information for

Reactive sputtering of SnS thin films using sulfur plasma and a metallic tin target: achieving stoichiometry and large grains

Daiki Motai[1], Issei Suzuki[1,*], Taichi Nogami[1], Takahisa Omata[1]

1. Institute of Multidisciplinary Research for Advanced Materials, Tohoku University, Sendai 980-8577, Japan

*Corresponding author: issei.suzuki@tohoku.ac.jp

Section S1. Optical properties of the fabricated SnS thin films

The transmittance (T) and reflectance (R) spectra of SnS thin films were measured using a spectrometer (Hitachi, U-4100) with a light incident angle of 5°, over the wavelength range of 250–1800 nm. For both measurements, a setup that maintained a consistent optical path length was used. The net transmittance spectrum (T^*) was obtained by eliminating the effects of reflection and interference, using the relation $T^* = T/(1–R)$. The absorption coefficient (α) as a function of photon energy was estimated using the equation $\alpha = \ln\,[(1–R)/T]/d$, where d is the film thickness determined from cross-sectional SEM observation. Figure S1 (a, b) shows the net transmittance spectra and the corresponding absorption coefficient spectra. For both the thin films deposited at T_{sub} = RT and 300 °C, steep absorption edges corresponding to the fundamental absorption due to the band gap are observed at 1.2-1.3 eV, which agrees well with reported values for the direct band gap of SnS [S1].

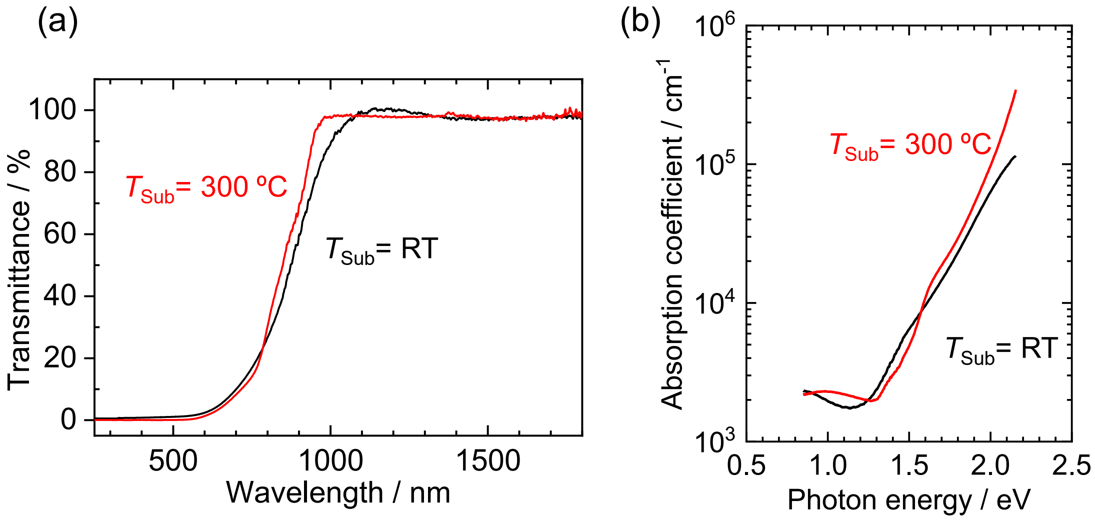

Figure S1. (a) Transmittance spectra and (b) corresponding absorption spectra of the SnS thin films fabricated at T_{Sub} = RT and 300 °C. In the absorption spectra, wavelength regions with transmittance values approaching 100% or 0% were excluded because the calculated absorption coefficient becomes less accurate under such conditions.

Section S2. Material balance and incorporation efficiency in reactive sputtering

When metallic Sn thin films were deposited by sputtering using a Sn target (RF_{Sn} = 5 W) without S-plasma or sulfur vapor, the deposition rate was 33 nm h^{-1} (Figure S2). In this case, assuming a relative density of 100% for metallic Sn thin films and sticking probability of 100% for atoms reaching the substrate surface, the flux of Sn atoms reaching the substrate surface was estimated to be 1.2×10^{17} atoms cm^{-2} h^{-1}.

The thickness of the SnS thin films fabricated in this study was 200 nm regardless of T_{Sub}, corresponding to a deposition rate 40 nm h^{-1}. Based on the assumption that the relative density of the SnS thin film is 100%, the incorporated Sn atoms as SnS thin film was 8.4×10^{16} atoms cm^{-2} h^{-1}. Thus, the fraction of Sn atoms reaching the substrate surface and being incorporated into the thin film was estimated to be approximately 70%.

When sulfur powder in the sulfur plasma source was heated at 100 °C, the weight loss of sulfur powder (supplied to the chamber) was 3-7 mg h^{-1}.[S2] Based on the actual deposition rate of SnS (40 nm h^{-1}, as described above), the amount of sulfur incorporated into the SnS thin films on the 40 × 40 mm^2 substrate was 0.07 mg h^{-1}. Therefore, 1–2% of the S-plasma supplied was incorporated into the SnS thin films. It is assumed that most of the remaining S-plasma either deposited outside the substrate or was exhausted from the chamber.

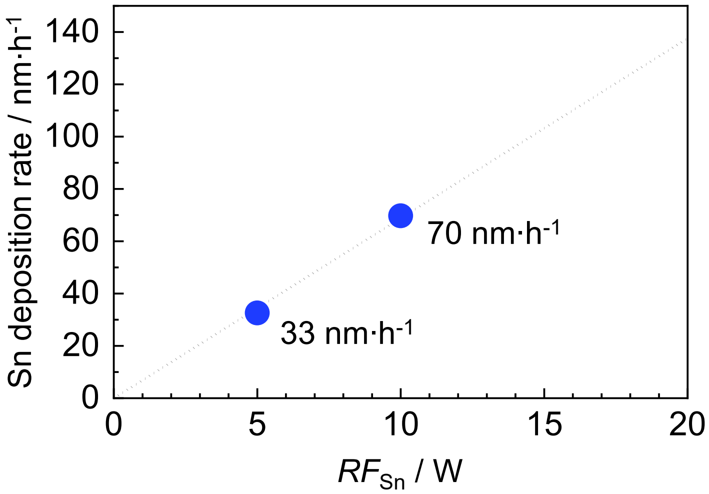

Figure S2. Deposition rate of metallic Sn as a function of RF power for a metallic Sn target deposited with *T-S* = 135 mm without S-plasma or sulfur vapor supply.

Section S3. Enlarged XRD profiles of the SnS thin films

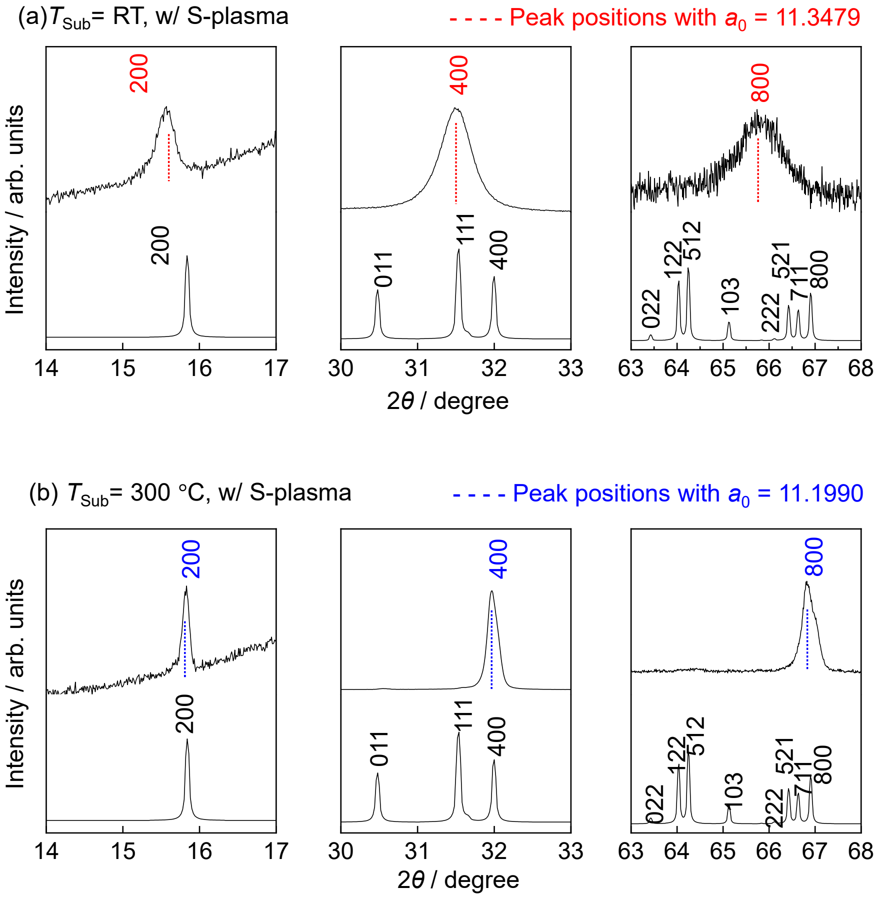

Figure S3. Enlarged XRD profiles of SnS thin films fabricated at (a) T_{Sub} = RT and (b) 300 °C. The vertical dotted lines in blue or red indicate the corresponding peak positions for the assumed lattice parameter or lattice plane spacing, which are shown in the upper right corner of each figure.

Section S4. In-plane XRD analysis

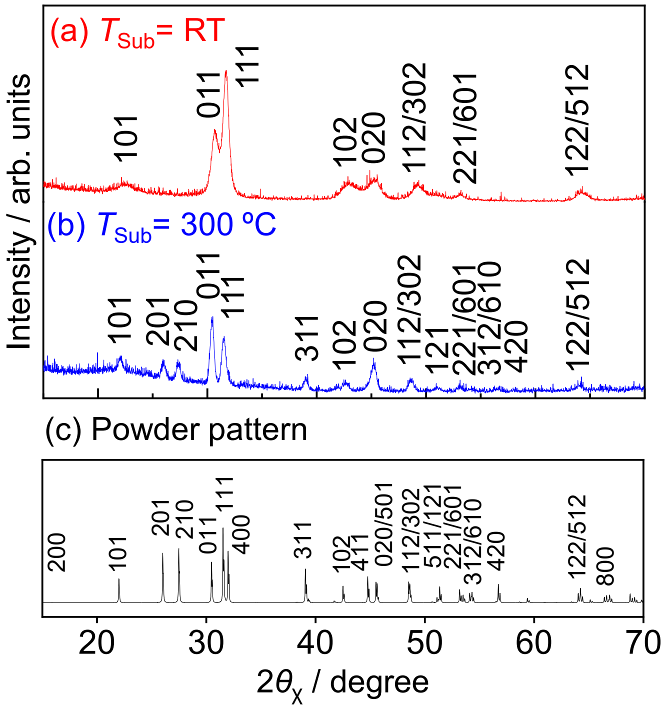

Figure S4. In-plane XRD profiles of thin films fabricated at (a) T_{Sub} = RT and (b) 300 °C with RF_{Sn} = 5 W and T-S = 135 mm, along with the powder pattern of SnS (ICSD#24376).[S3]

References for supplementary material

[S1] Kawanishi, I. Suzuki, T. Ohsawa, N. Ohashi, H. Shibata, and T. Omata, “Growth of large single crystals of n-type SnS from halogen-added Sn flux”, Cryst. Growth Des., 20, 5931–5939 (2020).

[S2] T. Nogami, I. Suzuki, D. Motai, H. Tanimura, T. Ichitsubo, and T. Omata, “Non-stoichiometry in SnS: How it affects thin-film morphology and electrical properties”, *APL Mater.*, 13, 031115 (2025)

[S3] S. Del Bucchia, J.C. Jumas, and M. Maurin, “Contribution à l’étude de composés sulfurés d’étain(II): affinement de la structure de SnS,” *Acta Crystallographica Section B*, B37, 1903 (1981).
